# Diffuse microvascular C5b-9 deposition is a common feature in muscle and nerve biopsies from diabetic patients

**DOI:** 10.1186/s40478-018-0512-6

**Published:** 2018-02-20

**Authors:** Paul C. Yell, Dennis K. Burns, Evan G. Dittmar, Charles L. White, Chunyu Cai

**Affiliations:** 1 Department of Pathology, UT Southwestern Medical Center, Dallas, Texas 75390 USA; 2 Long School of Medicine, UT Health San Antonio, San Antonio, Texas 78229 USA

**Keywords:** Peripheral neuropathy, C5b-9, Terminal complement complex, Diabetes, Nerve biopsy, Muscle biopsy

## Abstract

Terminal complement complex deposition in endomysial capillaries detected by a C5b-9 immunostain is considered a diagnostic feature for dermatomyositis. However, we found widespread microvascular C5b-9 reactivity in a substantial subset of muscle biopsies with denervation changes, and in nerve biopsies of peripheral neuropathies, particularly in patients with diabetes. It is unclear whether the presence of C5b-9 deposition signifies active immune-mediated vascular injury that requires immune suppression therapy. We retrospectively identified 63 nerve biopsies in patients with a documented history of diabetes, 26 of which had concomitant muscle biopsies, as well as 54 control nerve biopsies in patients without a documented diabetes history, 18 of which had concomitant muscle biopsies. C5b-9 immunostain was performed on all cases. 87% of the nerve biopsies and 92% of the muscle biopsies from diabetic patients showed microvascular C5b-9 reactivity, compared to 34% and 50% in non-diabetic patients. The differences were statistically significant (*p* < 0.0001 for nerve and *p* = 0.002 for muscle). The C5b-9 reactivity was generally proportional to the extent of microvascular sclerosis in diabetic patients, but unrelated to inflammation or vasculitis. C5b-9 deposition in micro-vasculature in both muscle and nerve is therefore a common feature in patients with diabetic neuropathies and may have diagnostic utility. Precaution needs to be taken before using muscle capillary C5b-9 reactivity as evidence of myositis.

**Electronic supplementary material:**

The online version of this article (10.1186/s40478-018-0512-6) contains supplementary material, which is available to authorized users.

## Introduction

C5b-9, also known as the terminal complement complex (TCC) or membrane attack complex (MAC), is a multimer derived from five complement components that mediates the cell killing effects of the complement cascade. Antibodies designed to detect this complex react to a neoantigen formed in C9 when this protein is incorporated into the large multimer. In general, endomysial capillary C5b-9 deposition has been considered to be supporting evidence of immune-mediated myositis and an indication for immune modulation therapy. Immunohistochemical detection of C5b-9 has long been used to support a diagnosis of dermatomyositis in muscle biopsies [7], in which condition deposition of C5b-9 in perifascicular endomysial capillaries has been regarded as a “specific” feature of dermatomyositis [1, 5]. More recently, perifascicular endomysial capillary deposition of C5b-9 has also been described in anti-synthetase syndrome-associated myositis [9] and necrotizing autoimmune myopathies with autoantibodies against signal recognition particle (SRP) and 3-hydroxy-3-methylglutaryl-coenzyme A reductase (HMGCR) [16]. In peripheral nerve biopsies, two clinical series have described C5b-9 deposition in endoneurial vessels in diabetic patients with peripheral neuropathy [6, 14]. However, it was not clear whether this feature would distinguish diabetic neuropathy from other etiologies, or indicated a need for immune modulation therapy.

During validation of this stain at our institution for myositis, it was noticed that a diffuse or patchy, non-perifascicular, endomysial capillary C5b-9 deposition pattern was not associated with myositis, but instead was very common in muscle with neurogenic changes. In particular, patients with a documented diabetes history who underwent muscle and sural nerve biopsies often showed diffuse C5b-9 deposition in both muscle endomysial capillaries and nerve endoneurial vessels. To date, microvascular C5b-9 deposition in both peripheral nerve and muscle has only been described in neuropathy with pipe-stem capillaries [13], dermatomyositis-associated sensory neuropathy [10], and neuropathy with serum anti-TS-HDS antibody [11]. These are rare, controversial entities only described in isolated case reports. Muscle endomysial capillary C5b-9 deposition has not been described in diabetic patients.

Given that diabetes is extremely common, it would be useful to know 1) the prevalence and extent of C5b-9 deposition in muscle and nerve microvasculature in patients with history of diabetes; 2) whether C5b-9 deposition indicates active vasculitis or inflammation in diabetic patients; and 3) whether C5b-9 deposition is a specific feature of diabetic peripheral neuropathy. We aimed to address these questions in this study by evaluating the C5b-9 staining pattern/intensity in nerve biopsies and their accompanying muscle biopsies in a cohort of patients with a documented history of diabetes who underwent biopsy to evaluate the nature of their peripheral neuropathy. Nerve and accompanying muscle biopsies from non-diabetic patients with peripheral neuropathies served as controls.

## Materials and methods

### Case selection

We retrospectively searched the SNOMED coded UT Southwestern Medical Center Neuropathology Database for the time period 1989 to 2016 and identified 63 cases of peripheral nerve biopsies in patients with a documented history of diabetes and sufficient stored frozen tissue. Twenty-six of those cases had concomitant muscle biopsies. An additional 54 consecutive cases of nerve biopsies from patients with an etiologic peripheral neuropathy diagnosis but no documented diabetes history were identified and serve as controls, 18 of those had concomitant muscle biopsy. A total of 117 nerve and 44 muscle biopsies from 117 patients were thereby included in this study.

Detailed clinical information, including neurology notes, history and physical examination notes, electromyography and nerve conduction study reports, and laboratory tests were available for 82 patients. Pathology reports were available for all patients.

### Histopathological evaluation

All case slides were individually reviewed by two pathologists (CC, PY) and evaluated for the following features (present or not present): fibrinoid necrosis, perivascular lymphocytic cuffing, and microvascular sclerosis. Microvascular sclerosis in nerve was assessed on toluidine blue stained plastic sections, defined by the presence of multiple endoneurial vessels with uniformly thickened walls that comprised 50% or more of their external diameter. A meeting was held to discuss results and reach a consensus for each of these features for each case.

### Immunohistochemistry of C5b-9

C5b-9 immunostain was performed on all 117 cases. Six-micrometer thick cryostat sections of frozen nerve or muscle tissue blocks underwent heat-induced epitope retrieval using CC1 (Ventana, Tucson, AZ), a tris-based buffer at pH 8–8.5, followed by immunohistochemical staining with a polyclonal mouse antibody to a neoepitope formed by poly (C9) in the terminal complement complex (Dako, M0777) diluted 1:500. Immunohistochemistry was performed on either the Ventana Benchmark XT or Ventana Benchmark Ultra automated immunostainer, using a Ventana UltraView Universal DAB Detection Kit.

All C5b-9 stained slides were independently reviewed by three pathologists (PY, DB, CC) in an arbitrary numerical order. The reviewers were blinded to each patient’s history, the final diagnosis, as well as slides prepared as a part of the routine diagnostic evaluation prior to this study.

### Statistical analysis

In order to evaluate the statistical significance of possible association between diabetes status versus C5b-9 grade, inflammation versus C5b-9 grade, and microvascular sclerosis versus C5b-9 grade, logistic regression analyses were performed to generate *p*-values using MedCalc Statistical Software version 16.1.2 (MedCalc Software bvba, Ostend, Belgium; https://www.medcalc.org; 2016). 2 × 2 contingency tables were also created to calculate sensitivity/specificity.

To evaluate inter-rater reliability of the three-tiered C5b-9 grading scheme detailed below, mean percent agreement values were generated by averaging the pairwise agreement percentages among the three pathologist reviewers for all muscle cases and all nerve cases considered separately, as well as within each tier of grading for muscle and nerve separately. Disagreement rates are expressed as (100 - agreement rate).

The data for C5b-9 grades among the three reviewers were then used to calculate Krippendorff’s alpha values for nerve and muscle cases separately, as a measure of overall reliability corrected for chance, using the online ReCalOIR tool [2]. While Cohen’s kappa is more commonly used to serve this analytical purpose, Krippendorff’s alpha is more appropriate here, primarily because our data are ordinal rather than nominal in nature, and also because alpha more naturally accommodates analyzing agreement among more than two reviewers. The interpretation of significance of Krippendorff alpha values recommended by Krippendorff himself is: α < 0.66 as unacceptable, 0.66 < α < 0.8 as borderline, and α > 0.8 as good reliability [8].

## Results

### Patients’ characteristics

Of the 63 patients with a documented diabetic history, the mean patient age was 56 (range 28–85 years) at the time of nerve biopsy. Six patients had juvenile onset type I diabetes, the remainder were adult onset type II diabetes. The majority of patients had a long history of diabetes and distal sensory polyneuropathy. The causes for nerve biopsy were heterogeneous and summarized in Table 1. Ten patients had a clinical diagnosis of diabetic amyotrophy (severe back pain and subacute onset proximal weakness). Nine patients had one or more systemic autoimmune conditions including lupus, rheumatoid arthritis, Sjogren’s syndrome, polyarteritis nodosa, sarcoidosis, paraneoplastic syndrome, and Crohn’s disease. Sural nerve biopsy was usually performed to rule out vasculitis or inflammation in patients with these autoimmune disorders. Four patients had a clinical diagnosis of possible or probable chronic inflammatory demyelinating polyneuropathy (CIDP). One patient each had lymphoma involving lumbosacral nerve roots, post-surgical neuropathy, and anti-GM1 motor neuropathy. The remaining patients had nerve biopsy to evaluate for a cause of progressive or worsening polyneuropathy.

**Table 1:** Summary of Peripheral Nerve Stain with C5b-9

Disease	Total (n)	0	1+	2+	1+ or 2+
Diabetic (Total)	63	7	28	28 /44.4%	56 /88.9%
Amyotrophy	10	0	1	9	10/100%
Autoimmune^a^	9	3	5	1	4/66.7%
CIDP	4	1	0	3	3/75%
SMPN	37	3	19	15	34/91.9%
Other^b^	3	0	3	0	3/100%
Non-Diabetic (Total)	29	22	5	2/6.9%	7/24.1%
CIDP	11	9	2	0	2/18.1%
GBS	2	1	0	1	1/50%
Hereditary	8	6	1	1	2/25%
Idiopathic PN	4	4	0	0	0/0%
Mononeuropathy multiplex	3	2	1	0	1/33.3%
Amyloidosis	1	0	1	0	1/100%
Unknown Diabetic Status (Total)	25	12	11	2/8.0%	13/52.0%
Amyloid neuropathy	6	5	1	0	1/16.7%
CIDP	6	3	3	0	3/50%
Hereditary	1	1	0	0	0/0
Mononeuropathy multiplex	4	0	3	1	4/100%
Idiopathic PN	3	2	0	1	1/33%
Microvascular sclerosis	5	1	4	0	4/80%

^a^: The “autoimmune” category under diabetic group includes: lupus, rheumatoid arthritis, Sjogren’s, polyarteritis nodosum, Crohn’s disease, sarcoidosis, paraneoplastic

^b^: The “other” category under diabetic group includes one case each of: lymphoma plexopathy, post-surgical neuropathy, and anti-GM1 motor neuropathy

Abbreviations: *CIDP* chronic inflammatory demyelinating polyneuropathy, *SMPN* sensory motor polyneuropathy, *GBS* Gillian Barré Syndrome, *PN* polyneuropathy

Of the 54 control patients, 29 were definitively non-diabetic patients at time of biopsy, supported by normal glucose/ hemoglobin A1c laboratory tests. The remaining 25 patients had an unknown diabetic status. The mean patient age was 51 years (range 6 months to 83 years) at the time of nerve biopsy. Their diagnoses included a variety of acquired and hereditary conditions including CIDP, Guillain-Barré syndrome (GBS), mononeuritis multiplex, amyloidosis, hereditary neuropathies (giant axon neuropathy, Charcot-Marie-Tooth disease, Leigh Syndrome) and idiopathic polyneuropathies (Table 1). Excluding hereditary neuropathies, the mean patient age was 58 (range 25 to 83 years), similar to those of the diabetic group.

### Interpretation of C5b-9 immunostain

**Fig. 1:**
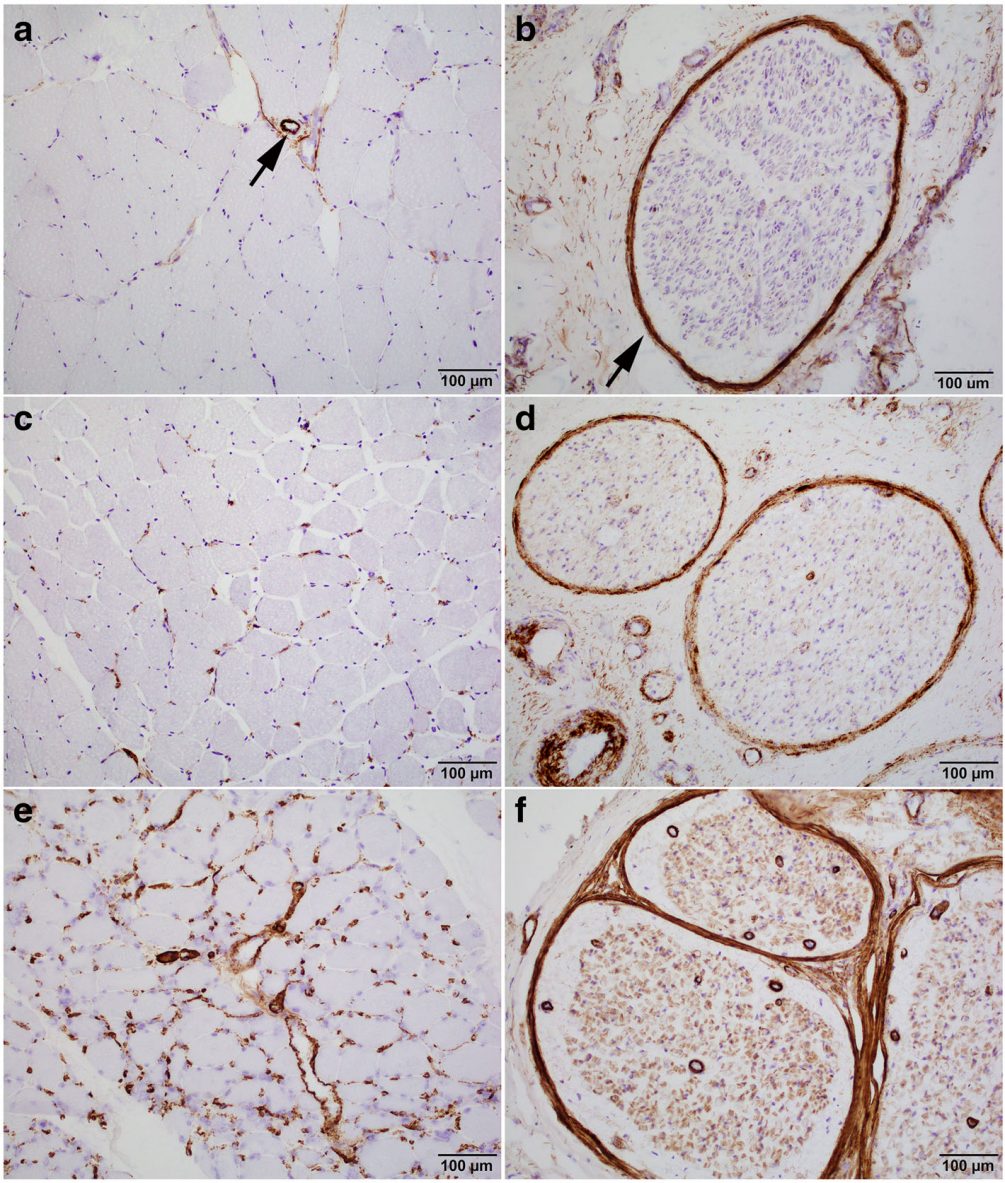
C5b-9 Grading Scheme in Muscle and Nerve**.** All muscle and nerve cases are scored 0, 1+ or 2+ based on endomysial (muscle) and/or endoneurial vessel (nerve) stains. **a** Muscle: 0: no capillary stain. Isolated weak granular stain allowed. Perimysial artery stain (arrow) was not considered pathological and served as internal control. **b** Nerve: 0: no endoneurial staining. Rare subperineurial or septal vessel stain or very weak granular vessel stain were still considered negative stain. Perineurium stain (arrow) was not considered pathological and served as internal control. **c** Muscle: 1+: Unequivocal circumferential capillary stain but focal or weak. **d** Nerve: 1+: Variable endoneurial vessel stain, majority weaker than perineurium. **e** Muscle: 2+: patchy or diffuse strong circumferential capillary stain. **f** Nerve: 3+: Circumferential stain in multiple vessels per fascicle, most equal to or stronger than perineurium

**Fig. 2:**
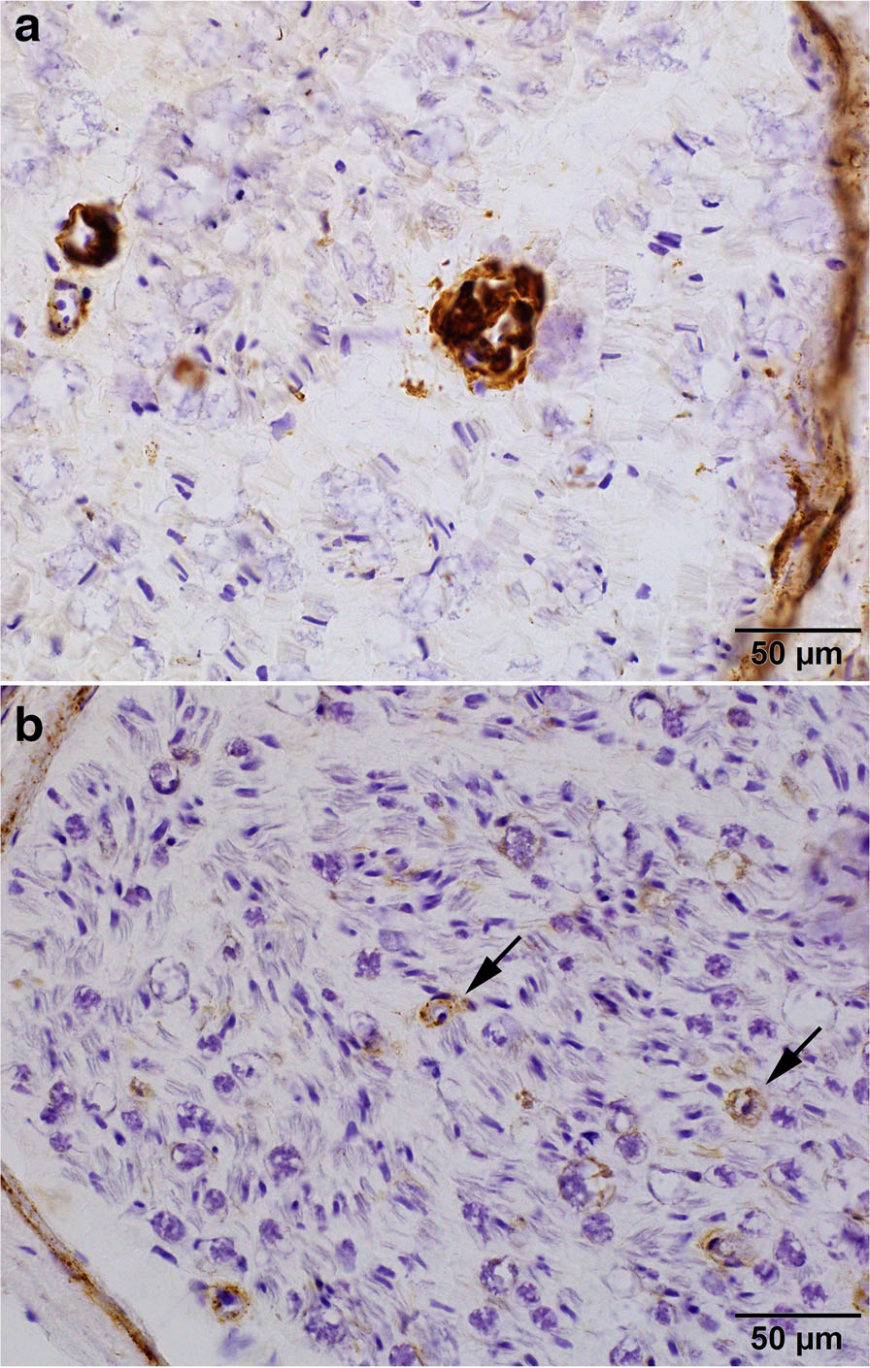
Amyloid and Schwann Cell Staining Patterns for C5b-9. **a** Non-capillary-type amyloid staining, graded as 0. **b** Schwann cell pattern of C5b-9 staining, graded as 0

Extent of C5b-9 reactivity was graded as strong (2+), focal/weak (1+) or absent (0) in endoneurial vessels and endomysial capillaries (Fig. 1, see legend for detailed criteria). Several pitfalls in the interpretation of C5b-9 were recognized. In cases with amyloid deposition, such deposits stained strongly with C5b-9, but in an irregular, granular pattern rather than the round, homogeneous, capillary wall pattern of other positive cases (Fig. 2a). Vessels without amyloid did not stain positively in these cases. Additionally, some GBS and CIDP cases may demonstrate Schwann cell C5b-9 reactivity (Fig. 2b, arrows), which has been described in the literature [4]. These cases typically have no C5b-9 reactivity in endoneurial vessels. Finally, strong C5b-9 reactivity was nearly always observed in the media of larger perimysial arteries (Fig. 1a, arrow) and the perineurium of peripheral nerves (Fig. 1b, arrow) in the vast majority of nerve and muscle biopsies in both diabetic patients and non-diabetic controls. Although the reason for those C5b-9 deposits remains unclear, they appear non-pathogenic and serve as reliable internal positive controls.

### Endoneurial microvascular C5b-9 deposition in diabetic and control patients

**Fig. 3:**
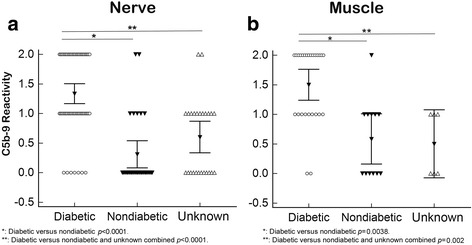
C5b-9 Grade Was Significantly Higher In The Diabetic Cohort Than Control. Multiple variables dot plots demonstrated higher C5b-9 grades in both nerve (**a**) and muscle (**b**) microvasculature in the diabetic cohort than the non-diabetic and unknown cohorts. The differences were statistically significant (*p* values were calculated by logistic regression analysis using 1+ as cut off)

Results of endoneurial microvascular C5b-9 reactivity are summarized in Table 1. A majority (88.9%) of nerves from diabetic patients showed either 2+ (44.4%) or 1+ (44.4%) endoneurial vessel C5b-9 reactivity. By contrast, less than a quarter (24.1%) of non-diabetic patients had 2+ (6.9%) or 1+ (17.2%) C5b-9 reactivity in endoneurial vessels. The difference was statistically significant using either 1+ (*p* < 0.0001) or 2+ (*p* < 0.0001) as cut off. Having either 1+ or 2+ C5b-9 reactivity in endoneurial vessels had a sensitivity of 88.9% and specificity of 75.9% for diabetes. In patients with unknown diabetic status, 8% had 2+ and 44% had 1+ C5b-9 reactivity. When comparing diabetic patients to combined non-diabetic and unknown group using 1+ as cut off, the difference was still statistically significant (*p* < 0.0001) (Table 1 and Fig. 3), but the specificity decreased to 63%.

### Endomysial microvascular C5b-9 deposition in diabetic and control patients

Microvascular C5b-9 staining (1–2+) was detected in 92.3% of muscle biopsies in diabetic patients, and 50% of non-diabetic patients (Table 2). The difference was statistically significant (*p* = 0.0038). When comparing diabetic patients to combined non-diabetic and unknown group, the difference was also statistically significant (*p* = 0.002). Interestingly, the majority of non-diabetic, CIDP cases were 0 intensity for C5b-9 in the nerve endoneurial vessels, but 1+ or 2+ in the accompanying muscle capillaries (Additional file 1). Hereditary and amyloidosis cases had minimal to no muscle capillary staining.

**Table 2:** Summary of Muscle Stain with C5b-9

Disease	Total (n)	0	1+	2+	1+ or 2+
Diabetic (Total)	26	2	9	15 /57.7%	24 /92.3%
Amyotrophy	6	1	0	5/83.3%	5/83.3%
SMPN	19	0	9	10/52.6%	19/100%
Lymphoma plexopathy	1	1	0	0	0
Non-Diabetic (Total)	12	6	5	1/8.3%	6/50%
Amyloid	1	1	0	0	0
CIDP	4	0	3	1	4/100%
Hereditary PN	6	5	1	0	1/16.7%
Idiopathic PN	1	0	1	0	1/100%
Unknown Diabetic status (Total)	6	3	3	0/0%	3/50%
Mononeuropathy multiplex	2	0	2	0	2/100%
CIDP	2	1	1	0	1/50%
Hereditary PN	0	0	0	0	0/0
Amyloidosis	2	2	0	0	0/0

Abbreviations: *CIDP* chronic inflammatory demyelinating polyneuropathy, *SMPN* sensory motor polyneuropathy, *PN* polyneuropathy

There was generally good concordance between the microvascular C5b-9 reactivity in muscle and nerve within the diabetic population. Of the nerves that had 1+ or 2+ endoneurial vascular reactivity, 23/25 (92%) of the accompanying muscle biopsies also showed 1+ or 2+ endomysial capillary C5b-9 reactivity. No such concordance was seen in the non-diabetic population. Having 1+ or 2+ C5b-9 microvascular reactivity in both the muscle and the nerve had a combined sensitivity of 88.4% and specificity of 83.3% for diabetes patients when comparing to the non-diabetic cohort.

### Diffuse and strong endoneurial microvascular C5b-9 reactivity is associated with microvascular sclerosis in diabetic patients, but not associated with inflammation

**Fig. 4:**
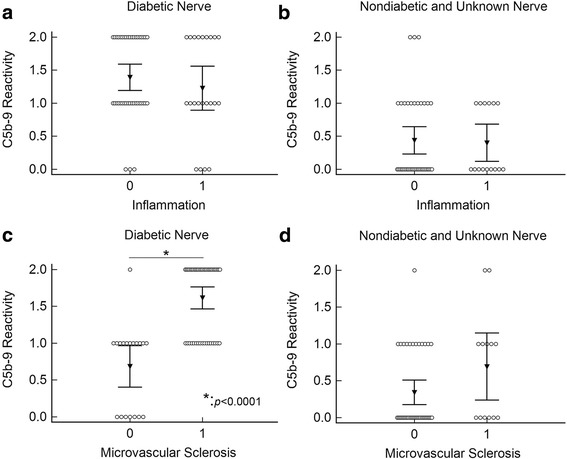
C5b-9 Grades Were Not Associated With Inflammation, But With Microvascular Sclerosis In Diabetic Patients. Multiple variable dot plots comparing endoneurial microvascular C5b-9 grade versus inflammation in the diabetic cohort (**a**) and the combined non-diabetic and unknown cohort (**b**) showed no association between C5b-9 and inflammation. Strong association was seen between C5b-9 and microvascular sclerosis in the diabetic cohort (**c**) (^*^: *p* < 0.0001 by logistic regression analysis using 1+ as cut off) but not in the combined non-diabetic and unknown cohorts (**d**)

**Fig. 5:**
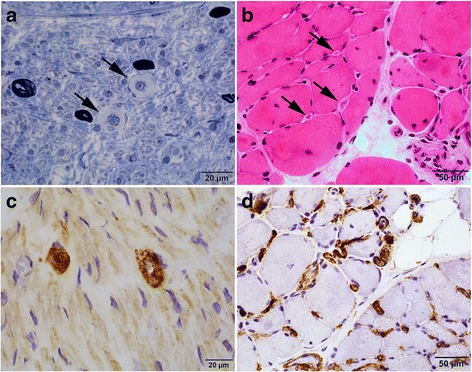
C5b-9 Grade Corresponds with Microvascular Sclerosis in Diabetic Patients. Toluidine blue stained plastic section of nerve (**a**) and H & E stained section of the muscle (**b**) showed prominent microvascular sclerosis (arrows) in a diabetic patient. C5b-9 immunostains in the same patient showed strong C5b-9 staining in the thick-walled vessels in both nerve (**c**) and muscle (**d**)

We next examined whether endoneurial microvascular C5b-9 deposition was associated with active vasculitis or inflammation. In the diabetic cohort, only one patient with polyarteritis nodosa had overt vasculitis with fibrinoid necrosis in a large epineurial artery. The endoneurial vessels showed 2+ C5b-9 reactivity. Another 21 nerve biopsies had nonspecific perivascular lymphocytic cuffing in the perineurium or endoneurium. Overall, there was no significant association between endoneurial microvascular C5b-9 reactivity and inflammation in the diabetic cohort, using either 2+ (*p* = 0.67) or 1+ (*p* = 0.20) C5b-9 reactivity as a cut off (Fig. 4a). Rather, cases with 2+ C5b-9 reactivity were nearly uniformly associated with prominent microvascular sclerosis (27/28 cases, Figs. 4c and 5a, c). Grade 0 C5b-9 reactivity cases were associated with minimal or no microvascular sclerosis (7/7). The association between C5b-9 reactivity and microvascular sclerosis was statistically significant in the diabetic cohort using either 2+ (*p* < 0.0001) or 1+ (*p* < 0.0001) as cut off. In non-diabetic and unknown status patients combined cohort, 16/54 nerve biopsies showed inflammation and 13/54 nerve biopsies showed unequivocal microvascular sclerosis. C5b-9 reactivity in endoneurial vessels was not associated with either inflammation (Fig. 4b) (*p* = 0.78 using 1+ as cut off; *p* = 0.16 using 2+ as cut off) or microvascular sclerosis (Fig. 4d) (*p* = 0.15 using 1+ as cut off; *p* = 0.11 using 2+ as cut off).

Microvascular sclerosis was not separately scored in muscle. However, most diabetic patients with 2+ C5b-9 showed prominently thickened endomysial capillaries that indented the adjacent myofibers (Fig. 5b, d).

### Inter-rater concordance

Mean percent agreement was generally good among the three reviewers, slightly higher for nerve (84%) than for muscle (73%) overall. Not surprisingly, when considered separately, cases graded as 1+ had distinctly lower agreement scores in both nerve and muscle than 0 or 2+ cases (Table 3). Krippendorff’s alpha coefficient showed excellent reliability for interpretation of C5b-9 grades in nerve (0.84) and borderline good reliability for muscle (0.76). Taken together, these results indicate that the C5b-9 scoring system detailed here is robust and reproducible insofar as we were able to test it with this case volume and number of reviewers.

**Table 3:** Inter-rater Concordance for C5b-9 Grading

	Mean % Agreement	Mean % Disagreement	Krippendorff’s Alpha
Nerve C5b-9			
Overall	84%	16%	0.842
0	95%	5%	
1+	70%	30%	
2+	92%	8%	
Muscle C5b-9			
Overall	73%	27%	0.76
0	94%	6%	
1+	53%	47%	
2+	79%	21%	

## Discussion

Diabetic polyneuropathy is a highly prevalent disease condition that affects approximately 30% of hospital-based patient populations, 20% of community-based patient populations, and 10% of the diabetic population identified by glucose tolerance screening [17]. The histologic features of diabetic peripheral neuropathy in sural nerve biopsies are non-specific and variable, and their presence may complicate the interpretation of a nerve biopsy when searching for other etiologies, such as immune-mediated demyelinating disorders.

In this study we showed that widespread microvascular deposition of C5b-9 in both muscle and nerve was characteristic of diabetic patients with peripheral neuropathy, with a combined sensitivity of 88.4% and specificity of 83.3% for the diabetic status when using 1+ as cut off. It should be emphasized, however, that C5b-9 deposition in endoneurial and muscle capillaries was not entirely specific for diabetic microvasculopathy. In our non-diabetic cohort, 2+ endoneurial vessel C5b-9 reactivity was seen in one patient with Charcot-Marie-Tooth type 2 neuropathy and one patient with GBS. 1+ or even 2+ muscle capillary reactivity was seen in a large proportion of non-diabetic CIDP patients. Additionally, C5b-9 strongly stained amyloid deposits, the Schwann cells in a subset of CIDP cases, the walls of large perimysial arteries in all adult muscle biopsies, and the perineurium of all adult nerve biopsies. The causes of C5b-9 deposition in these different conditions are probably heterogeneous and not all are necessarily pathological. Awareness of such diverse staining patterns is important for neuropathologists, neurologists, and rheumatologists alike to avoid diagnostic pitfalls.

There was no consistent correlation between active inflammation and C5b-9 deposition in endoneurial vessels among diabetic cases; nor was there any correlation between C5b-9 deposition and the different forms of diabetic neuropathies represented in our study population (distal symmetric sensory neuropathy, diabetic amyotrophy, and diabetes patients with superimposed CIDP or systemic autoimmune diseases). Rather, we found C5b-9 reactivity to generally correlate with the presence of microvascular sclerosis in diabetic patients. Interestingly, high rates of C5b-9 deposition have also previously been reported in the choriocapillaris of the eyes of patients with diabetic retinopathy [3] and the skin capillaries of diabetic patients [15], suggesting that microvascular C5b-9 deposition may be a widespread phenomenon in small vessels throughout the body. It has been hypothesized that glycation of the cell membrane protein CD59, which ordinarily is a specific inhibitor of C5b-9 formation [12], may underlie this pattern of C5b-9 reactivity. Another common cause for microvascular sclerosis is hypertension, which is not expected to be associated with C5b-9 deposition. Consistent with this notion, in our control cohorts, microvascular sclerosis was not associated with C5b-9 deposition. The microvascular C5b-9 deposition is most likely a chronic change influenced by long term glycemic control. Therefore, microvascular complement activation in diabetic patients, either in muscle or nerve, is probably not, by itself, an indication for immune modulation therapy.

The three-tiered C5b-9 scoring system detailed here is designed to be easy to use and reproducible. Among three reviewing pathologists, the concordance rate for the nerve is excellent with an overall alpha value of 0.84, and substantial agreement for the muscle with an overall alpha value of 0.76. This can prove quite useful in the assessment of muscle and nerve biopsies, particularly in the evaluation of biopsies submitted to a reference lab with limited provided clinical history. The presence of microvascular sclerosis and diffuse C5b-9 deposition in either muscle or nerve vessels should raise the concern of diabetes, a condition present in a significant proportion of the nerve biopsies received at our institution. Nerve biopsy is an invasive procedure and, with a few notable exceptions (e.g. vasculitis, inflammation, amyloid), one that often yields only limited information regarding the etiology of the neuropathy. We feel that routine performance of C5b-9 on nerve and accompanying muscle biopsy has the potential to identify patients with diabetic neuropathy, and directs further testing/treatment in these patients (e.g. diabetic investigation and glucose control). The finding of diabetic neuropathy does not, of course, exclude other etiologies, as superimposed conditions such as vasculitis, amyloidosis, CIDP and systemic autoimmune diseases were also identified in our cohort. A complete clinical history, including any history of diabetes should ideally always be provided in muscle or nerve biopsy cases, and performance of a muscle biopsy along with the nerve biopsy may significantly increase the diagnostic yield in both diabetic and non-diabetic patients with suspected neuropathies.

The major strengths of the current study are (1) the wide variety of peripheral nerve diseases that we evaluated which are frequently encountered in everyday neuromuscular pathology practice; (2) the use of semi-quantitative grading criteria for C5b-9 capillary positivity, which enabled calculations of sensitivity and specificity values; and 3) the correlation of C5b-9 reactivity with microvascular sclerosis in diabetic patients. The major limitations are 1) the retrospective nature of the study; 2) the muscle biopsies evaluated in this study were all accompanying biopsies along with sural nerve biopsy in evaluation for neuropathy, and thus may not represent routine muscle biopsies for myopathy; and 3) incompleteness of clinical record in approximately 30% of our subjects, whose biopsies were referred to our laboratory for evaluation from outside institutions.

## Conclusions

Diffuse, robust microvascular deposition of C5b-9 in both muscle and nerve biopsies is quite common in, though not entirely specific for, diabetic peripheral neuropathy. We demonstrated the novel finding of greater than 90% C5b-9 positivity in muscle biopsies of diabetic patients who underwent concurrent sural biopsy for neuropathy. The extent of positivity is correlated with severity of microvascular sclerosis, but not inflammation. Microvascular C5b-9 reactivity in diabetic patients can be a confounding factor when using this stain to support a diagnosis of dermatomyositis or Jo-1 myositis, though these entities typically have a perifascicular deposition pattern, rather than the diffuse/non-zonal pattern of diabetic patients. The three-tiered scoring system for C5b-9 that we developed for this study appears robust and reproducible.

## Additional file

Additional file 1: Clinical, epidemiological and pathological features of nerve and muscle biopsies included in this study. (XLSX 43 kb)
